# Maximizing reusability of learning objects through machine learning techniques

**DOI:** 10.1038/s41598-023-40174-w

**Published:** 2023-10-11

**Authors:** Meryem Amane, Mounir Gouiouez, Mohammed Berrada

**Affiliations:** 1 Artificial Intelligence, Data Science and Emergent Systems Laboratory, Sidi Mohammed Ben Abdellah University, Fez, Morocco; 2 PSCS Laboratory, Sidi Mohamed Ben Abdellah University, Fez, Morocco

**Keywords:** Engineering, Electrical and electronic engineering

## Abstract

Maximizing the reusability of learning objects through machine learning techniques has significantly transformed the landscape of e-learning systems. This progress has fostered authentic resource sharing and expanded opportunities for learners to explore these materials with ease. Consequently, a pressing need arises for an efficient categorization system to organize these learning objects effectively. This study consists of two primary phases. Firstly, we extract metadata from learning objects using web exploration algorithms, specifically employing feature selection techniques to identify the most relevant features while eliminating redundant ones. This step drastically reduces the dataset’s dimensionality, enabling the creation of practical and useful models. In the second phase, we employ machine learning algorithms to categorize learning objects based on their specific forms of similarity. These algorithms are adept at accurately classifying objects by measuring their similarity using Euclidean distance metrics. To evaluate the effectiveness of learning objects through machine learning techniques, a series of experimental studies were conducted using a real-world dataset. The results of this study demonstrate that the proposed machine learning approach surpasses traditional methods, yielding promising and efficient outcomes for enhancing learning object reusability.

## Introduction

The rapid development of distance-learning systems and the widespread availability of massive open online courses have profoundly influenced the sharing and reusability of learning objects. These learning objects represent valuable educational resources that play a pivotal role in catering to the specific learning needs of both teachers and students across various disciplines. However, despite the proliferation of learning objects, the efficiency of current e-learning environments in effectively utilizing these resources for reusability remains a challenge. Many e-learning platforms lack a systematic approach to classify learning objects, resulting in a suboptimal organization and limited opportunities for their multiple uses and reuses in different educational contexts. To address these limitations, this study employs cutting-edge Machine Learning algorithms for learning object classification. These advanced techniques offer the capability to extract patterns and insights from vast amounts of metadata associated with learning objects. By leveraging the power of Machine Learning, we can efficiently organize and categorize learning objects based on their unique characteristics, topic relevance, and educational objectives. In addition, we propose a novel approach that involves mining relevant information from the web to enhance learning object classification. Web mining techniques allow us to extract valuable data and insights from online sources, complementing the metadata-based classification process. By incorporating web mining, we gain a more comprehensive understanding of learning objects and can further refine their categorization. The proposed Machine Learning-based and web mining-driven classification approach aims to significantly enhance resource discovery and promote efficient knowledge dissemination within the e-learning environment. By accurately classifying learning objects, learners can easily access the most pertinent information, enabling them to select suitable materials that precisely align with their specific learning goals and interests.

Our research is two-fold in its objectives. Firstly, we seek to establish a comprehensive theoretical model for learning objects’ classification, combining the capabilities of Machine Learning algorithms with valuable insights derived from web mining. This integrated approach enables the systematic grouping and structuring of learning objects, thus facilitating their seamless integration into diverse educational contexts. Secondly, our study introduces a novel method for sharing learning objects, leveraging the benefits of web mining in conjunction with our advanced classification technique. This synergistic fusion allows for more intelligent and context-aware resource discovery, ensuring that learning objects are effectively disseminated and utilized across various e-learning platforms.

## Literature review

### Learning object

In recent years, there has been an intense debate about creating modern and effective digital teaching materials. These materials are often described as "Learning Objects". The main idea of the creation and schematization of learning objects as specific pedagogical tools is not far from the usual school materials, which are traditionally used by teachers in the classroom. What distinguishes the nature of learning objects from other types of documents is their digital form, their creation in a computer environment, but also their supply of special features that allow them to be searched in a specific repertoire. These objects, which are recognized in the international scientific community as learning object, present a significant diversity. A. Robertson^1^ attempted to schematize existing approaches: “For some, it is a numerical or non-numerical entity that can be indexed for learning purposes”. Learning object can be associated with content objects^2^, with educational objects^3^, with information objects, and with knowledge objects^4^. As M. Dodani^5^ encodes them, learning objects must have the following characteristics in order to function adequately at an individual level and in order to be easily and effectively used and transformed in different educational environments:

- They are small, self-contained units of learning that offer a concept, information, or a process. These entities are distributed after having been previously tested and evaluated.
- They are described by “metadata” that allows us to classify and search for them.
- They are combined with other learning objects to create complex educational entities, such as a set of concepts.

In practice, a learning object can be a web page, an image, a simulation, a test, or any other type of element involved in learning. Learning objects are not limited to courses or training content. A learning object can also refer to a procedure or guidelines to help the learner on his academic pathway.

**Figure 1:**
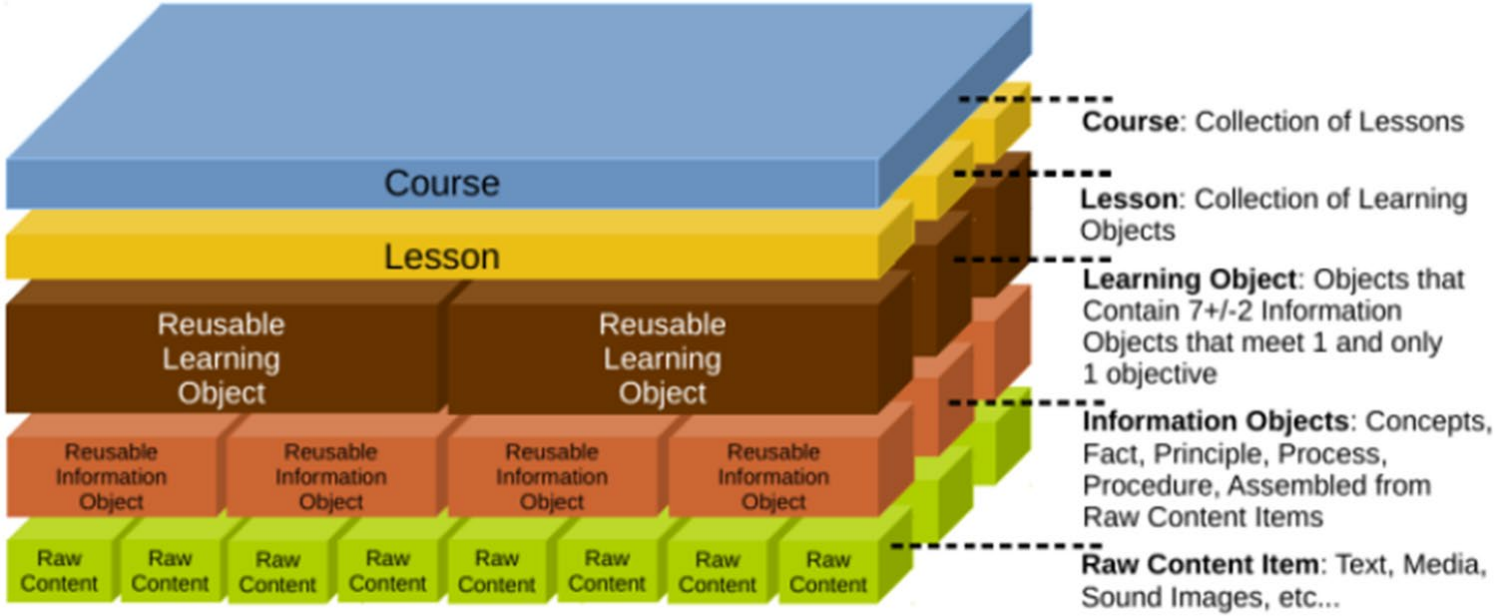
Representation of a content model for an LCMS with reusable learning objects (Working Group, 2002).

The Learning Technology Standards Committee (LTSC) has defined the concept of «learning objects» as “Any entity, digital or not, that can be utilised for learning, education, or training is characterised as a learning object”^6^, In his Ph.D. thesis, David Wiley analysed the design of learning objects and came up with his own definition, which was “any digital resource that can be reused to promote learning”^7^. In the organizational context, learning objects are generally defined in the terminology of the LMS or LCMS vendor. Chapman and Hall^8^, note that in this context, there is no coherent definition of a learning object; “…each of the companies using the learning object metaphor has its own defined relationship and characteristics for what constitutes a learning object”. An example of such a model is shown in Fig. 1.

This model illustrates the idea of a layered model that is often found in e-learning scenarios. Each layer represents a new level of abstraction, starting with a very concrete data object that encompasses exactly one element. In this model, the bottom layer consists of raw data, such as textual data, videos, presentations, etc. This data can be collected in a “Reusable Learning Object”. Chapman and Hall concluded their research by saying, “We hope to see better definitions and common standards for learning objects in the future”.

The learning object technology can be viewed at four different levels:

- The object technology itself, including the model of the reference used to label, or meta-label, the item.
- The technology related to the repository in which the objects are being collected, including the database technology and/or the content management technology of the learning object^8^;
- The technology for referential-related services, such as searching, browsing, previewing, and downloading tools.
- The technology to support the exchange or interaction of learning objects among systems and repositories

**Figure 2:**
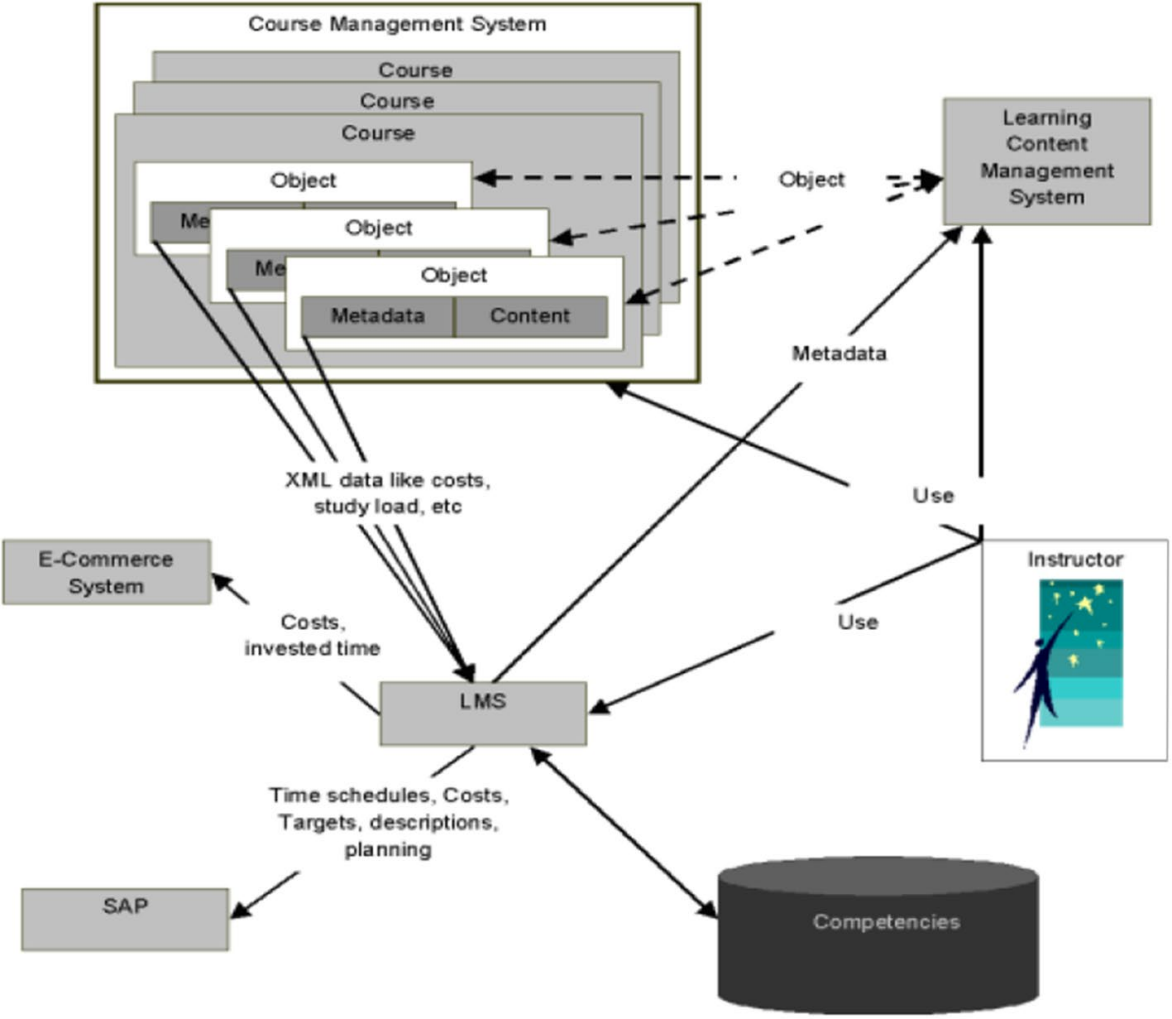
Learning objects resources in the e-learning system.

Learning objects have a number of functions in the current blended learning system. The prospective learning objects can be acquired through contracts with software developers, and also be procured through the exchange and sharing of digital materials that are being entered by course designers, course presenters, and course participants into the databases of learning content management systems, such as LMS, SAP, and CMS (course management systems)^9^ (Fig. 2).

### Classification of learning object in E-learning system

Many Numerous studies^10, 11^ have employed machine learning techniques to classify learning objects within e-learning systems. In^12^, the authors highlight the effectiveness of main classifiers used in e-learning systems and propose a model that combines decision trees, neural networks, and naive Bayesian methods into a unified module. Subsequent research in 2015 explored diverse resources and properties for the classification of learning objects. In 2019, authors^13^ introduced a novel concept of learning object classification based on Long Short-Term Memory (LSTM) and the Random Forest classification approach. The application of data mining methods in the e-learning process with web-based learning is outlined in this article. However, regarding Multi-Label Classification (MLC) paradigms, only nine papers in the literature have focused on developing learning objects in e-learning systems. These papers present various approaches to incorporate feature-label correlations into learning objects' metadata, aiming to improve accessibility and recommendations simultaneously. This work specifically concentrates on adapting formal single-label classifiers to handle multiple labels. Addressing the model for multi-label classification and ranking of learning objects, the authors of^10^ associate the concept of searching learning objects marked by Learning Object Metadata (LOM). The model provides a methodology that demonstrates the task of multi-label mapping of learning objects into different types of inquiries. In^11^, researchers identify learning items by comparing and contrasting four MLC systems. Furthermore, the authors of^14^ explore hierarchical multi-label categorization in the context of recommender systems. They propose a hierarchical multi-label metadata categorization using machine learning methods to enhance the search and classification of educational resources^15^. Moreover, this study contributes by providing a hierarchical multi-label learning object dataset in an appropriate format.

Table 1 presents a comprehensive review of significant research articles that have embraced MLC methods to advance learning object classification in e-learning systems. Through the adoption of diverse machine learning techniques, these studies have contributed significantly to enhancing the organization and accessibility of educational resources.

**Table 1:** Summary of the significant studies that applied MLC methods on the learning objects.

Perspective question	Title	Description
Davis et al. (2018)^16,17^	Multi-label green’s function criterion inspired transfer annotation system	This study proposes a multi-label classification system inspired by the Green's Function Criterion for transfer annotation
Wilson et al. (2019)^18,19^	Label enhancement manifold learning algorithm for multi-label image classification	The authors present a manifold learning algorithm for multi-label image classification, focusing on label enhancement
Johnson et al.^20^	Ensemble learning for learning object classification	The authors investigate the effectiveness of ensemble learning techniques in enhancing learning object classification accuracy
Williams et al.^21^	Hierarchical classification of learning objects for adaptive recommender systems	This study proposes a hierarchical classification approach for adaptive recommender systems to improve learning object recommendations
Brown et al.^22^	Learning object clustering using self-organizing maps	This research utilizes self-organizing maps for clustering learning objects, aiding in organizing and discovering relevant resources
Smith et al.^23^	Utilizing deep neural networks for learning object categorization	This research explores the application of deep neural networks for the categorization of learning objects in e-learning systems

## Current challenges

Based on the historical and present research contexts, several questions and challenges arise, laying the foundation for research inquiries in this study. The utilization of learning objects relates to understanding what is being reused, along with the level of detail and granularity involved. This encompasses the aspects of duplicating, connecting, blending, and publishing the material. Additionally, questions emerge concerning the various actors involved, determining who reuses what. The question of where material is being reused considers not only the exchange of material among individuals and organizations but also the distinctions among different learning situations. Moreover, the content product development lifecycle plays a role in defining when a material becomes available for reuse. These aspects can be further explored from a more organized and collaborative perspective. Table 2 demonstrates how the common learning object questions can be viewed through their structural and relational perspectives, providing valuable insights into the research. Machine learning techniques can be employed to analyze and uncover patterns and relationships within this data, enabling a deeper understanding of learning object reuse and facilitating more efficient and targeted approaches to enhance learning experiences.

**Table 2:** General questions related to learning objects.

Perspective question	Description
Why?	What is the purpose of the reuse? Why would humans need to invest time and effort during the different stages of a learning object's life cycle?
Who?	Who is engaged in the Reusability process?
What?	What is the gradation and the type of material being reused?
Where?	Where does reuse fit in terms of systems?

All of these questions, as well as other common questions, will be asked in different ways, and will discuss learning objects at different points in their development.

### Novelty of our research

In this study, we address several current challenges pertaining to the utilization of learning objects in the context of educational resources and content development. Our research seeks to explore and provide insights into various aspects of learning object reuse, which have not been extensively investigated in previous works. One of the primary novelties of our research lies in understanding the intricacies of learning object reuse, including the level of detail and granularity involved. We delve into questions surrounding what is being reused, how it is duplicated, connected, blended, and published, thereby shedding light on the intricacies of content repurposing. Another novel aspect we address is the identification of the different actors involved in learning object reuse. By determining who reuses what, we gain a deeper understanding of the stakeholders and their roles in the content reuse process. Furthermore, our study explores the geographical and situational dimensions of learning object reuse. We not only investigate the exchange of materials among individuals and organizations but also examine the distinctions between different learning contexts. This provides a comprehensive perspective on the spatial distribution of learning object reuse. Additionally, we introduce the element of timing into the investigation by considering the content product development lifecycle. Our research analyzes when a material becomes available for reuse, contributing to a better understanding of the temporal aspects of content reuse. To achieve these insights, we propose the application of machine learning techniques to analyze and uncover patterns and relationships within the data. This innovative approach allows us to gain a deeper and more structured understanding of learning object reuse, paving the way for more efficient and targeted strategies to enhance learning experiences. By addressing these current challenges and offering fresh perspectives on learning object reuse, our study contributes significantly to the advancement of knowledge in the field of educational content development and learning optimization.

## Research work

In this article, our first thought started with the idea that semantically similar terms are used in similar contexts. This arises the question of how we can manipulate e-learning resources like courses, videos, and pedagogical support in a flexible manner. Additionally, how can we explore and reuse these resources efficiently in different areas? For the first question, we have used object learning philosophy, in which every resource can be analysed and schematized according to IEEE learning objects standardisation, such as metadata, taxon, identifier, etc. To explore this metadata, our interest is focused on FCM Clustering Algorithm**,** web data mining and machine learning algorithms. For the second question, we thought of using MLC in order to reuse them in different contexts.

### Web data mining

The Web Data Mining concept was defined in 1996 as “a new generation of computational theories and tools to assist humans in extracting useful information (knowledge) from the rapidly growing volumes of digital data”^24^. The fundamental objectives of web data mining can be summarized as follows: they bring into the fore front invisible information, they take into account the volume of web data, they also transform the massive amount of web data into expert knowledge, and they provide valuable knowledge to the users despite the numerous attempts to characterize this field. The term “web data mining process” is frequently used with a combination of different techniques from various disciplines, including data analysis, artificial intelligence, and machine learning^25^. A typical process of web data mining can be described in three successive steps: data preparation or pre-processing data, discovering patterns and analyzing patterns. The pre-processing phase includes cleaning operations needed for the metadata of learning object normalization. In other words, it reduces the data dimension by implementing different tasks, allowing the elimination of extra information like stop words, double adjectives, etc. In the second phase, all information already prepared in the pre-processing step deals with the extraction methods in which all data is labelled using machine learning algorithms. Finally, in the analyzing process, the set of appropriate patterns will be presented by degree of similarities^26^.

### Fuzzy C-means clustering algorithm

Clustering is a statistical analysis method that is used to organize raw data into homogeneous silos^27^. Within each cluster, data is grouped according to a common characteristic. The clustering tool is an algorithm that measures the proximity between each element based on defined criteria. The purpose of clustering algorithms is to make sense of data and extract value from large amounts of structured or unstructured data. These algorithms allow the segmentation of data based on its properties or functionality and help to group them into different clusters based on their similarities. There are two types of clustering: hierarchical clustering and non-hierarchical clustering^28^. This explains that Fuzzy C-Means clustering is an unsupervised non-hierarchical clustering algorithm that tries to partition a finite collection of elements into a collection of fuzzy clusters with respect to some given criterion^29^. The algorithm of fuzzy c-mean clustering can be summarized as follows: In the beginning, it fixes the value of c (number of clusters) and selects a value of m (generally m takes a value between 1.25 and 2), and initializes the partition matrix Dij, then compute cluster centers, which will be repeated until the maximum convergence is reached.

### Multi-label classification approach

MLC is an automatic process that uses analysis techniques in order to label objects and classify them by topic. This approach uses a supervised learning method where a feature may be connected with multiple labels. It is opposed to single-label classification when each feature is associated only with a single class (label). Furthermore, MLC is widely used in real-world problems such as bioinformatics, e-commerce, and so on. Due to their efficiency with the huge size of data and the difficulties of assigning a single label to objects, MLC plays an important role in the process of learning object classification. However, few and insufficient studies have explored the MLC problem in the e-learning area.

## Methodology

**Figure 3:**
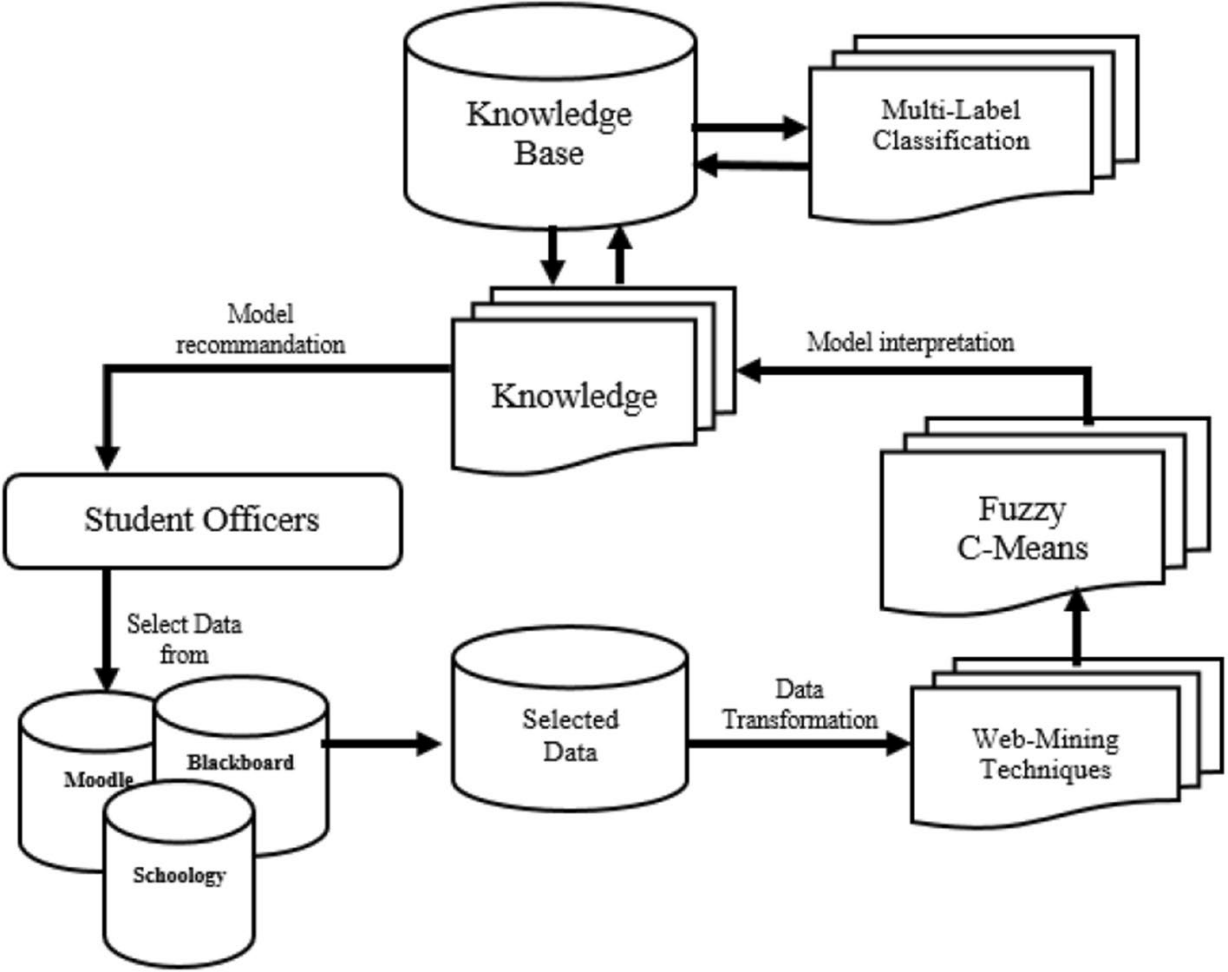
Illustration of our design with MLC Algorithm combined with FCM and web-mining.

Our methodology is designed with two distinct stages, as depicted in Fig. 3. The first stage, known as the learning phase, plays a pivotal role in generating a comprehensive list of learning objects. This list is generated through a combination of web mining techniques and similarity analysis performed by Fuzzy C-Means (FCM) algorithm. During the learning phase, our algorithm effectively extracts relevant learning objects from various web sources using web mining techniques. These techniques enable the algorithm to gather a diverse set of learning materials, encompassing a wide range of topics and domains. Subsequently, the FCM algorithm comes into play to assess the similarity between these extracted learning objects, categorizing them based on their relatedness. In the second stage, our algorithm proceeds with classifying the learning objects listed in the first phase. This classification is executed by evaluating the degree of similarity between these objects. The algorithm efficiently labels these learning objects with multi-label classification, thereby enabling efficient object reuse and facilitating personalized recommendations for learners. By adopting this two-stage approach, we aim to achieve two primary objectives. Firstly, we anticipate an enhancement in the similarity of the terms analyzed and extracted by the web mining techniques. This improvement will contribute to a more refined and precise selection of learning objects, ensuring their relevance and accuracy for learners. Secondly, our methodology focuses on optimizing the metadata of each learning object. By fine-tuning the metadata, we aim to facilitate the seamless usage and retrieval of learning objects, streamlining the learning experience for users.

### Human or animal participants

There were no experiments conducted on humans, nor were human tissue samples used in this study.

## Evaluation and discussion

### Corpora summary

For this research study, a diverse set of data has been collected from different companies, encompassing presentation, practice, and conceptual models of learning objects across various categories. The datasets were obtained from Moodle, a free and open-source learning platform with specific solutions for educational needs; Blackboard, a widely recognized digital learning platform with core learning management features; and Schoology, a platform dedicated to providing educators with essential tools for lesson design, student communication, and collaboration. The inclusion of datasets from these prominent companies enriches the study’s potential to explore learning object classification using machine learning techniques comprehensively. It allows for a robust analysis and optimization of learning object reusability in e-learning systems, enhancing the overall impact and relevance of the research findings.

### Experimental procedure and performance measure

To evaluate the performance of our approach, we used a set of indicators in the form of mathematical rules, such as classification accuracy (1), precision (2), recall (3), and F1-measure (4). These indicators are generally used to examine the performance of any proposed system compared to the existing ones. The following are descriptions of these indicators:

#### Classification accuracy

Classification accuracy shows how many of the predictions are correct.

1 $$Accuracy=\frac{Number of correct predictions}{Number of all predictions}$$

In some situations, accuracy serves as a good measure, while in others, it appears insufficient. For instance, a prediction accuracy of 94% indicates that 94 out of 100 samples were successfully anticipated without prior knowledge of which tasks were properly predicted.

#### Precision and recall

By going beyond classification accuracy, precision, and recall measures, they give us a clearer picture of how to evaluate models. The task and our objectives will determine which one we should favour.

Precision measures how good our model is when the prediction is positive.

2 $$Precision=\frac{TP}{TP+FP}$$

With:

TP (True positive): Predicting positive object as positive (ok)

FP (False positive): Predicting negative object as positive (not ok)

Recall measures how good our model is at correctly predicting positive classes.

3 $$Recall=\frac{TP}{TP+FN}$$

With:

FN (False negative): Predicting positive class as negative (not ok)

F1 measure is calculated as follows:

4 $$F1 measure=\frac{2.Precison.Recall}{precision+Recall}$$

### Dimensionality reduction

The selection criterion is mainly aimed at selecting the most relevant model that suggests the most appropriate learning objects. For this, classification techniques are very beneficial, both for the management systems that contain these objects and for the learners, who will benefit from a simple and fast search. In addition to this, with a good classifier, the initial data will be transformed into a new dimension by ignoring the massive data that is not appropriate.

In our article, we will evaluate the validity of our approach by comparing its indicators of performance (PR: precision, RE: recall, and F1-measure) to the traditional machine learning algorithms for classification and suggestion like SVM and Naive Bayesian. For this concern, we will use Python as a software platform, which has all these machine learning algorithms already programmed. In our approach, we will first test the performance of the data with SVM, and then we will move to test the same data with SVM combined with multi-labeled classification and FCM. To confirm the precision of our suggested approach, the same data was used with NB (Naive Bayesian) and NB combined with Multi-labeled classification and FCM. The results appear in Table 3 show that our approach performs better for the classification of learning objects compared to other learning algorithms in different corpora. The computed results of different classifiers in this table confirm that the results of SVM combined with the fuzzy logic clustering method and multi-ladled classification for data reduction are generally better than NB and its variants.

**Table 3:** Comparison of different machine learning methods using FNN and FCM.

Model	DataSet	Precision (%)	Recall (%)	F1 Score (%)
SVM	Moodle	90.03	53.04	87.5
SVM	Blackboard	94.52	67.87	89.10
SVM	Schoolary	89.45	51.87	89.01
NB	Moodle	84.19	45.90	77.3
NB	Blackboard	83.22	58.43	79.5
NB	Schoolary	87.16	49.67	81.23
Random forest	Moodle	91.25	56.72	89.33
Random forest	Blackboard	92.87	70.11	90.21
Random forest	Schoolary	88.91	54.79	88.67
SVM + Fuzzy C-means	Moodle	92.07	58.81	91.4
SVM + Fuzzy C-means	Blackboard	95.52	72.87	90.01
SVM + Fuzzy C-means	Schoolary	89.89	57.43	90.98
NB + Fuzzy C-means	Moodle	87.37	49.56	83.1
NB + Fuzzy C-means	Blackboard	85.01	64.94	79.9
NB + Fuzzy C-means	Schoolary	87.34	51.25	90.01
Random forest + Fuzzy C-means	Moodle	93.11	61.34	92.87
Random forest + Fuzzy C-means	Blackboard	93.76	74.12	91.02
Random forest + Fuzzy C-means	Schoolary	90.45	59.87	89.22

The analysis of the results reveals the performance of different machine learning models for learning object classification in three diverse datasets: Moodle, Blackboard, and Schoolary. Support Vector Machine (SVM) consistently exhibits remarkable accuracy across all datasets. For instance, in the Moodle dataset, SVM achieves a high precision of 90.03%, recall of 53.04%, and an F1 score of 87.5%. This indicates that SVM can accurately identify relevant learning objects and minimize false positives, making it a reliable choice for learning object classification. Random Forest, another powerful model, stands out in the Blackboard dataset, where it achieves the highest F1 score of 90.21%. In this dataset, Random Forest strikes a balance between precision and recall, effectively classifying learning objects with diverse attributes. Naive Bayes, while slightly lower in performance compared to SVM and Random Forest, still shows reasonable effectiveness. For example, in the Schoolary dataset, Naive Bayes achieves a precision of 87.16%, recall of 49.67%, and an F1 score of 81.23%. While not as high as SVM and Random Forest, Naive Bayes remains competitive in learning object classification. Furthermore, integrating Fuzzy C-Means with SVM or Random Forest models leads to notable improvements in some cases. For instance, in the Moodle dataset, SVM combined with Fuzzy C-Means achieves a precision of 92.07%, recall of 58.81%, and an impressive F1 score of 91.4%. This highlights the efficacy of incorporating fuzzy clustering techniques to enhance classification accuracy and increase the number of correctly identified learning objects.

## Conclusion

In conclusion, this research paper highlights the paramount importance of machine learning in addressing the complex problem of learning object classification in e-learning systems. By harnessing the power of advanced machine learning techniques, namely multi-label classification and Fuzzy C-Means, the study aims to revolutionize the recommendation process for learning objects, making them more accessible and reusable for learners. The adoption of the multi-label classification approach allows learning objects to be applied across various learning contexts, resulting in improved top n-recommendations and a more personalized learning experience. Machine learning plays a pivotal role in this research, as it enables the extraction of valuable patterns and insights from vast amounts of metadata associated with learning objects. By leveraging the capabilities of machine learning algorithms, the study can efficiently organize and categorize learning objects based on their unique characteristics, topic relevance, and educational objectives. This not only streamlines the handling and sharing of learning objects but also enhances their discoverability for learners, enabling them to access the most relevant and suitable materials aligned with their specific learning goals. Additionally, the integration of Fuzzy C-Means further enhances the classification process by calculating object similarity and reducing data volume by eliminating irrelevant instances. This fine-tuning of the classification process enhances the overall efficiency and accuracy of the system, ensuring that learners receive meaningful and contextually appropriate recommendations. The research's comprehensive evaluation using data from renowned platform systems (Moodle, Blackboard, and Schoology) further validates the superiority of the proposed machine learning-driven approach over traditional algorithms like SVM and NB. The evaluation metrics, including precision, recall, and F1 measure, confirm the machine learning approach's efficacy in achieving precise and efficient learning object classification.

## Data Availability

The datasets used and/or analysed during the current study available from the corresponding author on reasonable request.
